# Survey of Edible *Amanita* in Northern Thailand and Their Nutritional Value, Total Phenolic Content, Antioxidant and *α*-Glucosidase Inhibitory Activities

**DOI:** 10.3390/jof9030343

**Published:** 2023-03-10

**Authors:** Jaturong Kumla, Nakarin Suwannarach, Yuan S. Liu, Keerati Tanruean, Saisamorn Lumyong

**Affiliations:** 1 Research Center of Microbial Diversity and Sustainable Utilization, Chiang Mai University, Chiang Mai 50200, Thailand; 2 Department of Biology, Faculty of Science, Chiang Mai University, Chiang Mai 50200, Thailand; 3 Biology Program, Faculty of Science and Technology, Pibulsongkram Rajabhat University, Phitsanulok 65000, Thailand; 4 Academy of Science, The Royal Society of Thailand, Bangkok 10300, Thailand

**Keywords:** *Amanitaceae*, biological properties, edible mushroom, nutrition value, phylogeny, tropical area

## Abstract

Edible wild mushrooms are extremely popular among consumers and are highly valued for their potential economic benefits in northern Thailand. In this present study, a total of 19 specimens of edible *Amanita* were collected during investigations of wild edible mushrooms in northern Thailand during the period from 2019 to 2022. Their morphological characteristics and the phylogenetic analyses of the internal transcribed spacer (ITS) and partial large subunit (nrLSU) of ribosomal RNA, RNA polymerase II second-largest subunit (*rpb2*) and partial translation elongation factor 1-alpha (*tef-1*) indicated that the collected specimens belonged to *A*. *hemibapha*, *A*. *pseudoprinceps*, *A*. *rubromarginata*, *A*. *subhemibapha*, and *Amanita* section *Caesareae*. This is the first report of *A*. *pseudoprinceps* and *A*. *subhemibapha* from Thailand. Full descriptions, illustrations and a phylogenetic placement of all specimens collected in this study are provided. Subsequently, the nutritional composition and total phenolic content, as well as the antioxidant and *α*-glucosidase inhibitory activities, of each species were investigated. The results indicate that the protein contents in both *A*. *pseudoprinceps* and *A*. *subhemibapha* were significantly higher than in *A*. *hemibapha* and *A*. *rubromarginata*. The highest total phenolic content was found in the extract of *A*. *pseudoprinceps*. In terms of antioxidant properties, the extract of *A*. *pseudoprinceps* also exhibited significantly high antioxidant activity by 2,2-azino-bis(3-ethylbenzothiazoline-6-sulfonic acid (ABTS), 2,2-diphenyl-1-picrylhydrazyl (DPPH) and ferric reducing antioxidant power (FRAP) assays. However, the extract of *A*. *rubromarginata* had the lowest total phenolic content and level of antioxidant activity. Additionally, *α*-glucosidase inhibitory activity varied for different *Amanita* species and the highest level of *α*-glucosidase inhibitory activity was found in the extract of *A*. *pseudoprinceps*. This study provides valuable information on the nutrient content, phenolic content and the antioxidant and *α*-glucosidase inhibitory potential of edible *Amanita* species found in northern Thailand.

## 1. Introduction

The genus *Amanita* Pers. was first introduced in 1797 by Persoon [1] with *A*. *muscaria* (L.) Lam. as the type species. This genus is one of several large genera with approximately 650 species distributed throughout tropical, subtropical and temperate regions around the world [2,3,4,5,6]. *Amanita* is a member of the family *Amanitaceae*, order *Agaricales* [3,4,5,6]. Generally, *Amanita* is characterized by agaricoid basidiomata having free lamellae, white spore prints, hyaline and smooth basidiospores, as well as the presence of volval remnants (universal veil) and the presence of annulus (partial veil) on the stem [1,7,8,9]. Currently, taxonomic studies have divided this genus into three subgenera (*Amanita* subg. *Amanita*, *Amanitina,* and *Lepidella*) and eleven sections based on multi-gene phylogenetic analyses [4,10,11]. Most of the *Amanita* species are known to be ectomycorrhizal fungi that form mutualistic symbioses with more than ten families of plants (including *Betulaceae*, *Caesalpiniaceae*, *Casuarinaceae*, *Dipterocarpaceae*, *Fabaceae*, *Myrtaceae*, *Pinaceae*, and *Salicaceae*) and are known to grow on the ground in forests [3,4,9,12]. However, the *Amanita* species in *Amanita* sect. *Lepidella* have been reported as saprobes that grow in grasslands [4,9,13,14,15]. Notably, *Amanita* contains both edible and lethal species. The most toxic species are in *Amanita* sect. *Phalloideae* [e.g., *A. exitialis* Zhu L. Yang & T.H. Li, *A. phalloides* (Vaill. ex Fr.) Link, *A. verna* Bull. ex Lam. and *A. virosa* Bertill.], while most of the edible species belong to *Amanita* sect. *Caesareae* [2,4,16,17,18]. The most famous edible *Amanita* species are *A. caesarea* (Scop.) Pers., *A. chepangiana* Tulloss & Bhandary, *A. flammeola* Pegler & Piearce, *A. franchetii* (Boud.) Fayod, *A. fulva* Fr., *A. hemibapha* (Berk. & Broome) Sacc., *A. jacksonii* Pomerl., *A. manginiana* Har. & Pat., *A. loosii* Beeli, *A. pseudoporphyria* Hongo, *A. princeps* Corner & Bas, *A. rubescens* Pers., *A. tuza* Guzmán, *A. vaginata* (Bull.) Lam., and *A. zambiana* Pegler & Piearce [3,4,5,6,17,18,19,20].

**Figure 1:**
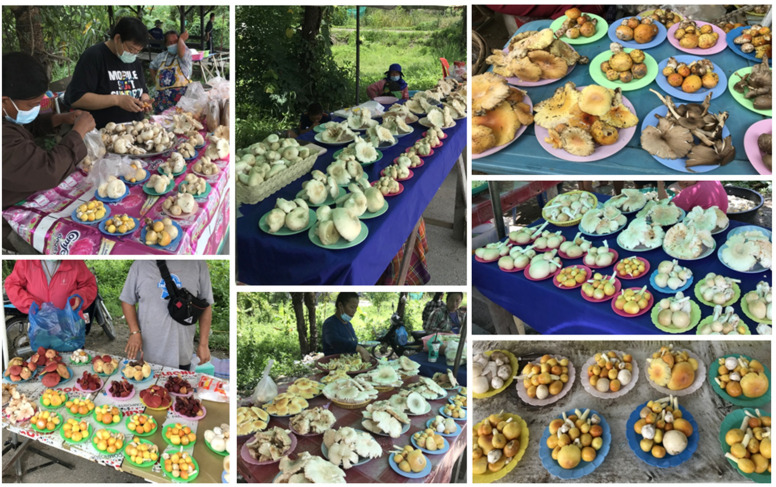
The local and roadside markets in northern Thailand sell a variety of edible wild mushrooms, including *Amanita* species. Photo credit by Kumla, J.

Several edible wild mushrooms are known to be a good source of essential dietary minerals, nutrients, and vitamins, which makes them an important source of food for humans [17,20,21]. These mushrooms have also been recognized as a source of many bioactive compounds (e.g., immunomodulatory compounds, phenolic compounds, polysaccharides, terpenoids and tocopherols) that exhibit various beneficial biological activities including anticancer, antidiabetic, anti-inflammatory, antimicrobial, antioxidant, cholesterol-reducing, immunomodulatory and neuroprotective properties [22,23]. Additionally, ethnomycologists have recorded vital information on the relevant consumption patterns and applications of wild edible mushrooms for medicinal purposes [24,25]. Thailand, a Southeast Asian country, has many species of edible wild mushroom that are particularly abundant during the rainy season (mid-May to October) each year. Generally, wild edible mushrooms are collected by local farmers for consumption and sale in local, roadside or city markets (Figure 1).

Preliminary investigations of edible wild mushrooms in northern Thailand have revealed the existence of many genera, e.g., *Amanita*, *Astraeus*, *Boletus*, *Cantharellus*, *Lactarius*, *Phlebopus*, *Russula*, and *Termitomyces* [26,27,28]. Edible *Amanita* species are the most popular variety of edible wild mushrooms in northern Thailand because of their palatable texture and flavor. However, the number of lethal and edible *Amanita* species that have been found in Thailand has remained a controversial issue due to the absence of comprehensive herbarium reference material, accurate descriptions and available molecular data [29].

During our ongoing studies of edible wild mushrooms in northern Thailand, we have collected specimens of edible *Amanita* species from natural forests, roadsides and local markets. Therefore, the present study aimed to identify the collected specimens based on their morphological characteristics and multi-gene phylogeny using the sequence data of ITS, nrLSU, *rpb2*, and *tef-1*. A full description, color photographs, illustrations and a phylogenetic tree of the collected specimens are provided. Moreover, the nutritional composition, total phenolic content, and antioxidant and *α*-glucosidase inhibitory activities of collected edible *Amanita* were investigated.

## 2. Materials and Methods

### 2.1. Sample Collection

The edible *Amanita* were surveyed and collected from natural forests, roadsides and local markets in Chiang Mai and Lamphun Provinces in northern Thailand during the rainy seasons of the years 2019 to 2022. Basidiomata were kept in plastic boxes and taken to the laboratory. Specimens were dried in a hot air oven at 45 °C until they were completely dry. After that, the dried specimens were kept in a plastic Ziplock bag and deposited in the Herbarium of Sustainable Development of Biological Resources (SDBR-CMU), Faculty of Science, Chiang Mai University, Thailand.

### 2.2. Identification of the Edible Amanita

#### 2.2.1. Morphological Observations

Fresh specimens were used to describe macromorphological data. Color names and codes were followed by Kornerup and Wanscher [30]. The dried specimens were examined for micromorphological data. Dried specimens were mounted in 5% aqueous KOH, Melzer’s reagent, or 1% aqueous Congo red solution. A light microscope (Nikon Eclipse Ni U, Tokyo, Japan) was used to examine micromorphological features. Each microscopic structure’s size data were derived from at least 50 measurements using the Tarosoft (R) Imaging Frame Work program. The terminology for microscopic features followed Largent et al. [31] and Bas [32]. Basidiospore statistics are expressed as (a–) b–c (–d), where ‘a’ and ‘d’ are the extreme values and ‘b–c’ is the range comprising 90% of all values. The Q value represents ratio of the length divided by the width of each basidiospore and Qm is the average Q of all specimens ± standard deviation.

#### 2.2.2. DNA Extraction, Amplification, Sequencing, and Phylogenetic Analyses

A Genomic DNA Extraction Mini-Kit (FAVORGEN, Ping-Tung, Taiwan) was used to extract DNA from fresh tissue of each specimen. The ITS, nrLSU, *rpb2,* and *tef-1* regions were amplified by polymerase chain reaction (PCR) using ITS5/ITS4 [33], LR0R/LR5 [34], Am6F/Am7R [35], and EF1-983F/EF1-1567R [36] primers, respectively. The PCR for these four domains was performed in separate PCR reactions on a peqSTAR thermal cycler (PEQLAB Ltd., Fareham, UK). The PCR programs of ITS, nrLSU, *rpb2*, and *tef-1* genes were established by following the methods employed by Liu et al. [15] and Cai et al. [36]. PCR products were directly sequenced by the Sanger sequencing method at 1st Base Company (Kembangan, Malaysia).

Sequence analysis was performed by a similarity search using the BLAST program available at NCBI (http://blast.ncbi.nlm.nih.gov, accessed on 12 November 2022). Sequences from this study, previous studies, and the GenBank database were selected and listed in Table 1. The combined dataset of ITS, nrLSU, *rpb2*, and *tef-1* was used for the phylogenetic analysis. MUSCLE [37] was used to perform multiple sequence alignments, and BioEdit v. 6.0.7 [38] was used to make any necessary improvements. Maximum likelihood (ML) and Bayesian inference (BI) methods were used to construct phylogenetic trees. The best substitution models were GTR+I+G for ITS, nrLSU and *tef*-*1* and HKY+I+G for *rpb2* from the Akaike Information Criterion (AIC) in jModeltest 2.1.10 [39]. The GTRCAT model with 25 categories was subjected to ML analysis using RAxML v7.0.3 and 1000 bootstrap replications [40,41]. MrBayes v3.2.6 [42] was used for the BI analysis, which evaluated the posterior probabilities (PP) using Markov chain Monte Carlo sampling (MCMC). Six simultaneous Markov chains were run from random trees for one million generations and trees were sampled every 100th generation. The first 25% of trees were discarded and the remaining trees were used for calculating PP value in the majority rule consensus tree. FigTree v1.4.0 [43] was used to visualize the tree topologies.

### 2.3. Nutritional Analysis

A total of six samples of edible *Amanita* (SDBR-CMUNK0775, SDBR-CMUNK0776, SDBR-CMUNK0780, SDBR-CMUNK0853, SDBR-CMUNK0855, and SDBR-CMUNK0857) obtained in this study were used in the analyses of nutrition, antioxidant, and *α*-glucosidase inhibitory activities because their dry weights were sufficient for testing. A Waring blender (New Hartford, CT, USA) was used to grind each dried sample. The nutritional composition (including ash, carbohydrate, fat, fiber, and protein) of each dried sample was determined using a method developed by the Association of Official Analytical Chemists (AOAC) [44] at the Central Laboratory Company Limited (Chiang Mai, Thailand).

### 2.4. Preparation of Mushroom Extracts

Ten grams (10 g) of each ground mushroom sample was extracted with 100 mL of absolute ethanol at 25 °C and 150 rpm for 24 h, as described by Kaewnarin et al. [45]. After that, each extract was placed in an ultrasonic bath (Elma Transsonic Digital, Singen, Germany) at 60 °C for 3 h. Whatman’s No. 1 filter paper was used to filter the samples. The residue was then re-extracted twice with absolute ethanol as mentioned above. The ethanolic extract was then dried using rotary evaporation at 40 °C. The extract was dissolved in 100 mL absolute ethanol and kept at 4 °C until further determination.

### 2.5. Determination of Total Phenolic Content

The method of Thitilertdecha et al. [46] was modified slightly to determine the total phenolic content. Folin-Ciocalteu reagent at 0.5 mL was mixed with 2.5 mL deionized water and 0.25 mL mushroom extract. After 5 min, 0.5 mL of Na_2_CO_3_ (20% *w*/*v*) was added. The mixture was incubated for 1 h in the dark at 25 °C. Measurements of absorbance at 760 nm were used to investigate the total phenolic content. The total phenolic content of the samples was calculated using a standard curve of gallic acid. Results were expressed as milligrams of gallic acid equivalents per gram of dry weight (mg GAE/g dw). Each sample extract was analyzed in five replicates. 

### 2.6. Antioxidant Assay

#### 2.6.1. ABTS Scavenging Assay

The procedure of Re et al. [47] with slight modifications was used to determine the 2,2-azino-bis(3-ethylbenzothiazoline-6-sulfonic acid) (ABTS) radical scavenging activity. The stock solution of ABTS cation chromophore was prepared by facilitating a reaction between 100 mL of 2.45 mM K_2_S_2_O_8_ and 100 mL of 7.0 mM ABTS solution. The solution was kept for 16 h in a dark place at room temperature. The ABTS solution was diluted with phosphate buffer (50 mM, pH 7.4) before use to yield an absorbance value of 0.70 ± 0.2 at 734 nm. A quantity of 2.9 mL of ABTS solution was mixed with 0.1 mL of each sample extract. The mixtures were incubated in the dark for 30 min at room temperature. A mixture of absolute ethanol and ABTS solution was used as the control. After incubation, the absorbance of each mixture was measured spectrophotometrically at 734 nm. Trolox was used as a reference compound. The ABTS scavenging activity was expressed as the Trolox equivalent antioxidant capacity per gram of dry weight (TE/g dw). Each sample extract was subjected to five replications. 

#### 2.6.2. DPPH Scavenging Assay

The method developed by Gülçin et al. [48] was used to determine the 2,2-diphenyl-1-picrylhydrazyl (DPPH) radical scavenging activity. Initially, 1.5 mL of the 0.1 mM DPPH solution in methanol was combined with 0.5 mL of the sample extract. The mixtures were incubated at room temperature in the dark for 30 min. Then, the absorbance of each mixture was determined using spectrophotometry at 517 nm. Trolox was used as a reference compound. The DPPH scavenging activity was expressed as the TE/g dw. Five replicates were performed for each sample extract.

#### 2.6.3. FRAP Assay

The method developed by Li et al. [49] was used to determine the ferric reducing antioxidant power (FRAP) activity. The FRAP reagent was prepared using a mixture containing 20 mL of 20 mM ferric (III) chloride, 10 mM 2,4,6-tripyridyl-s-triazine solution in 20 mL of 40 mM HCl, and 5 mL of 300 mM acetate buffer (pH 3.6). A quantity of 1.5 mL of FRAP reagent and 1.4 mL of acetate buffer (300 mM, pH 3.6) were mixed with 0.1 mL of each sample extract. Then, the mixture was incubated in the dark for 30 min at room temperature. Trolox was used as a reference compound. A mixture of absolute ethanol and FRAP solution was used as the control. After incubation, the absorbance of each mixture was measured spectrophotometrically at 593 nm. Trolox was used as a reference compound and the FRAP value was expressed as the TE/g dw. Five replicates were performed for each sample extract.

### 2.7. Determination of α-Glucosidase Inhibitory Activity

The procedure of Oki et al. [50] was modified to prepare the *α*-glucosidase solution from rat intestinal acetone powder. A quantity of 3 mL of 0.9% NaCl solution was mixed with 100 mg of intestinal acetone powder (Sigma-Aldrich Chemical Co., Saint Louis, MO, USA), homogenized by sonication, and stored in an ice bath. The enzyme mixture was centrifuged at 4 °C for 30 min at 8000 rpm. The supernatant was maintained in an ice bath and directly subjected to inhibitory assay. The *α*-glucosidase inhibitory assay was followed the procedure of Tanruean et al. [51] with some modifications. Each extracted sample (10 μL) was mixed with *α*-glucosidase solution (30 μL) and incubated at 37 °C for 15 min. Later, 70 μL of 37 mM D-maltose was then added and incubated at 37 °C for 15 min. The reaction was stopped after 10 min in boiling water. A glucose oxidase assay was used to determine the released glucose concentration of the reaction mixture. The peroxidase-glucose oxidase (PGO) reagent (900 µL) containing 1 capsule of PGO enzymes to 100 mL of water and 1.6 mL of o-dianisidine solution was added to the reaction mixture and it was then mixed for 30 min at 37 °C in a water bath. The absorbance of *α*-glucosidase activity was measured at 450 nm. The percentage of inhibition was calculated according to the formula: Percentage of inhibition = (Ao−As/Ao) × 100, where Ao is the absorbance of the control and As is the absorbance of the mixture containing the test compound. Acarbose (a standard synthetic inhibitor of *α*-glucosidase) was used for standard compound. Each sample extract was analyzed in five replicates.

### 2.8. Statistical Analysis

Statistical differences between treatments were assessed using one-way analysis of variance (ANOVA) with the SPSS program version 16.0 for Microsoft Windows. Significant differences at the *p* < 0.05 level were determined using Tukey’s test. The Pearson correlation coefficients (*r*) of the total phenolic content with antioxidant and *α*-glucosidase inhibitory activities of extract samples were analyzed using the SPSS program at a significance level of *p* < 0.05.

## 3. Results and Discussion

### 3.1. Sample Collection and Morphological Observations

In this study, a total of 19 edible *Amanita* specimens were obtained (Table 2). These specimens were initially classified into four *Amanita* species, namely *A. hemibapha* (4 specimens), *A. pseudoprinceps* (7 specimens), *A. rubromarginata* (4 specimens), and *A. subhemibapha* (4 specimens), based on their morphological characteristics. Subsequently, multi-gene phylogenetic analysis further confirmed their identification. 

### 3.2. Phylogenetic Analyses

**Figure 2:**
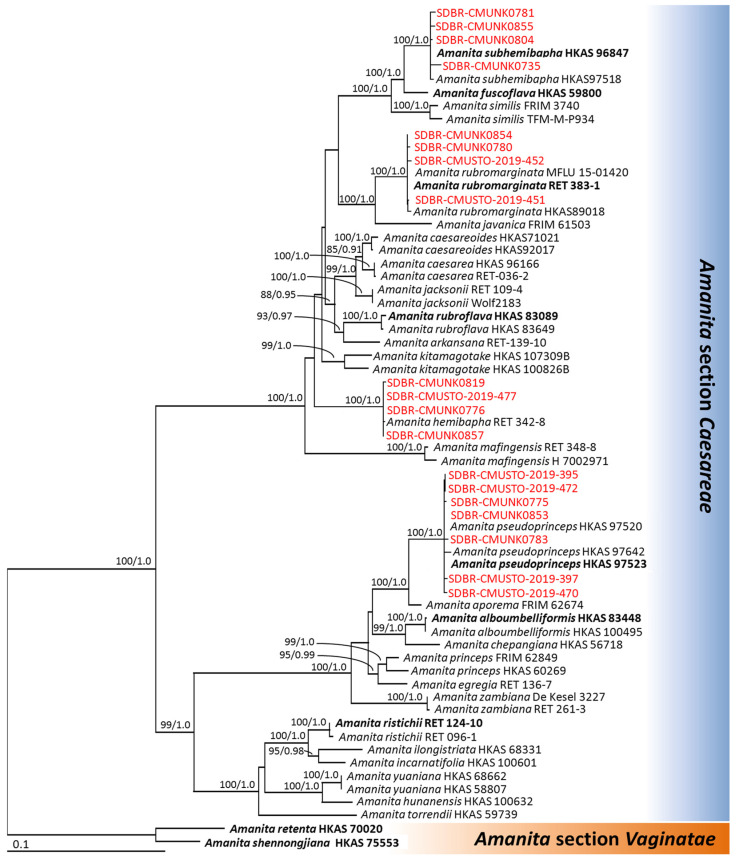
The phylogenetic tree derived from maximum likelihood analysis of 64 specimens of the combined ITS, nrLSU, *rpb2,* and *tef-1* genes. The tree is rooted with *A. retenta* and *A. shennongjiana*. Numbers above branches are the bootstrap percentages (left) and Bayesian posterior probabilities (right). Bootstrap values ≥ 75% and Bayesian posterior probabilities ≥ 0.90 are shown. The scale bar displays the expected number of nucleotide substitutions per site. Sequences derived in this study are shown in red. Type species are shown in bold.

The aligned dataset of the combined ITS, nrLSU, *rpb2*, and *tef-1* sequences consisted of 2831 characters including gaps (ITS: 1–903, nrLSU: 904–1676, *rpb2*: 1677–2316, and *tef-1*: 2317–2831). The matrix had 1192 different alignment patterns and 23.41% gaps or undetermined characters. A final ML Optimization Likelihood value of −15972.8506 was the best-scoring RAxML tree. For BI analysis, the final average standard deviation value of the split frequencies at the end of the total MCMC generations was calculated as 0.00723. The topology of the phylogenetic trees from the ML and BI analyses were similar. A phylogenetic tree obtained from the ML analysis is represented in Figure 2. Our phylogenetic tree was constructed with the aim of having similar outcomes to previous phylogenetic studies [4,52,53,54]. The phylogenetic tree consisted of 62 specimens of *Amanita* sect. *Caesareae* and two specimens of *Amanita* sect. *Vaginatae* (the outgroup). The phylogenetic tree clearly separated the 19 specimens obtained in this study into four species clades, namely *A. hemibapha* (4 specimens), *A. pseudoprinceps* (7 specimens), *A. rubromarginata* (4 specimens), and *A. subhemibapha* (4 specimens) in *Amanita* sect. *Caesareae* with high supported values (BS = 100% and PP = 1.0).

### 3.3. Morphological Descriptions

#### 3.3.1. ***Amanita hemibapha*** (Berk. & Broome) Sacc., Syll. Fung. (Abellini) 5: 13 (1887) (Figure 3)

*Basidioma* medium-sized. *Pileus* 6–12 cm diam., plano-convex with the center slightly depressed, orange-red (6A6–8) to lemon yellow (3B8) at center, and becoming vivid yellow (3A7–8) to pale yellow (3A3–4) towards the margin; universal veil on pileus white patch; margin striate (0.3 R), non-appendiculate; context 5 mm wide, white (1A1) to yellowish white (2A2), unchanging. *Lamellae* free, crowded, cream white (1A1–2); lamellulae truncate. *Stipe* 7–10.5 × 0.5–1.5 cm, cylindrical, covered by light yellow to vivid yellow (2A5–8) fibrous squamules; context broadly fistulose, white (1A1). *Bulb* absent. *Universal veil on stipe base* saccate, membranous, up to 4 cm high, white (1A1). *Partial veil* subapical, fragile, vivid yellow (3A7–8). 

**Figure 3:**
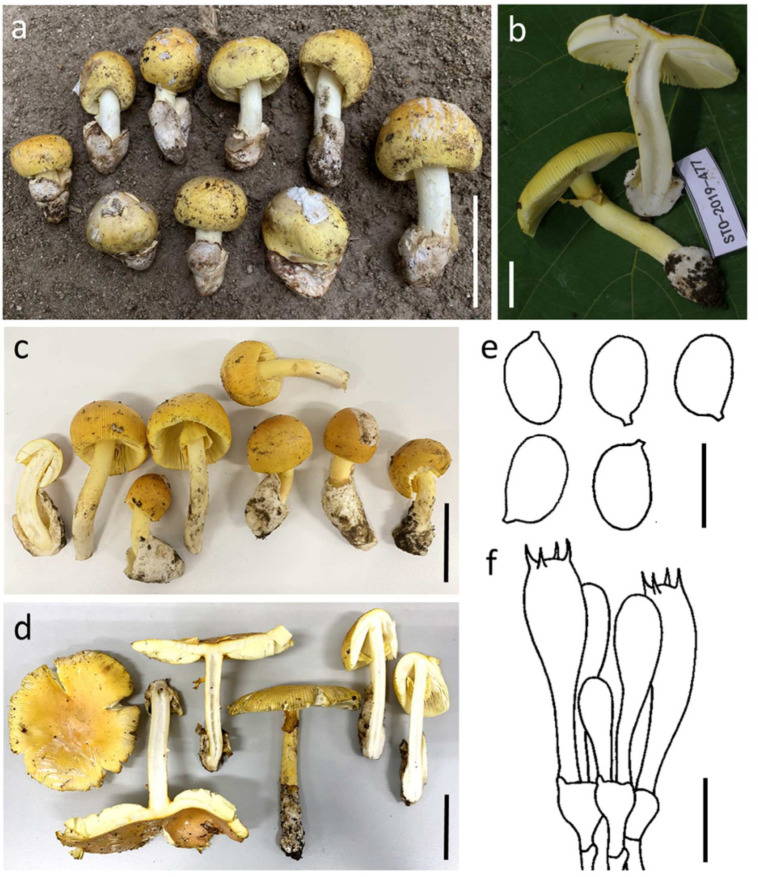
*Amanita hemibapha* SDBR-CMUNK0776 (**a**), SDBR-CMUSTO-2019-477 (**b**), SDBR-CMUNK0857 (**c**) and SDBR-CMUNK0819 (**d**). Basidiomata (**a**–**d**). Basidiospores (**e**). Basidia (**f**). Scale bars: (**a**,**c**,**d**) = 5 cm; (**b**) = 1 cm; (**e**) = 10 μm; (**f**) = 15 μm.

*Lamellar trama* bilateral, divergent; mediostratum 25–40 μm wide, filamentous hyphae abundant, 2–8 μm wide; clavate to ellipsoidal inflated cells 70–98 × 20–23 μm; vascular hyphae scarce. *Subhymenium* 20–35 μm thick in 2–3 layers, with subglobose to ellipsoidal or irregular cells, 6–25 × 5–15 μm. *Basidia* 32–50 × 8–12 μm, clavate, 4-spored with sterigmata 3–5 μm long; clamps present at base. *Basidiospores* (7.0–) 7.5–11.0 (–12.0) × 5.5–7.0 μm, Q = 1.23–1.64 (–1.71) μm, Qm = 1.44 ± 0.13, broadly ellipsoid to ellipsoid, sometimes elongate, inamyloid, hyaline, thin-walled, smooth; apiculus small. *Lamellar edge* sterile; filamentous hyphae 1–5 μm wide, hyaline, thin-walled; inflated cells, with subglobose, ovoid to ellipsoidal, 14–40 × 12–30 μm, single and terminal or in chains of 2–3, hyaline, thin-walled. *Pileipellis* 60–110 μm thick; 2-layered, upper layer 15–35 μm thick, filamentous hyphae 1–5 μm wide, weakly gelatinized, branching, thin-walled, hyaline; lower layer 50–85 μm thick, filamentous hyphae 2–7 μm wide, branching, thin-walled, hyaline to light yellow; vascular hyphae rare. *Inner surface of universal veil on stipe base* filamentous hyphae dominant 1–9 μm wide, hyaline to light yellow, thin-walled, branching; inflated cells, with subglobose, pyriform to clavate, 30–85 × 20–54 μm, hyaline, thin-walled; vascular hyphae rare. *Outer surface of universal veil on stipe base* similar to structure of inner part, but presenting more abundant inflated cells. *Stipe trama* longitudinally acrophysalidic; filamentous, undifferentiated hyphae 1–12 μm wide, thin-walled, frequently branching; acrophysalides 65–190 × 25–65 μm, thin-walled; vascular hyphae rare. *Partial veil* filamentous hyphae very abundant, 2–9 μm wide, hyaline, thin-walled; inflated cells scarce to locally abundant, globose, subglobose to clavate, 22–70 × 9–25 μm, hyaline to light yellow, thin-walled; vascular hyphae rare. *Clamp connections* present in all tissues of basidioma.

Habitat: Solitary to scattered on soil in tropical deciduous forests dominated by *Dipterocarpus* and *Shorea*. 

Distribution: known from China [3,4,8,10], India [55,56], Sri Lanka [57], and Thailand [5], this study.

Specimens examined: Thailand, Chiang Mai Province, Mae Taeng District, 19°07′45″ N 98°45′51″ E, alt. 1421 m, 9 August 2019, Yuan S.L., STO-2019-477 (SDBR-CMUSTO-2019-477); Doi Saket District, 18°53′2″ N 99°9′17″ E, alt. 343 m, 26 July 2020, Kumla J. and Suwannarach N., CMUNK0819 (SDBR-CMUNK0819), 11 August 2020, Kumla J. and Suwannarach N., CMUNK0857 (SDBR-CMUNK0857); Lumphun Province, Mueang District, Chiang Mai University Haripunchai Campus, 18°30′10″ N 99°8′25″ E, alt. 400 m, 25 July 2020, Suwannarach N., CMUNK0776 (SDBR-CMUNK0776).

Remarks: The remarkable features of *A. hemibapha* include the fact that this species has a reddish yellow or orange-red tone in the center of its pileus that becomes vivid yellow or pale yellow towards the edges. This species is also known to have a yellow annulus. *Amanita hemibapha* was firstly reported from Sri Lanka, and then found in China, India and Thailand [3,4,5,8,10,55,56,57]. Morphologically, *A. hemibapha* is easily confused with *A*. *caesareoides* Lj. N. Vassiljeva, *A*. *kitamagotake* N. Endo & A. Yamada, *A*. *rubroflava* Y.Y. Cui, Q. Cai & Zhu L. Yang and *A*. *subhemibapha* Zhu L. Yang, Y.Y. Cui & Q. Cai. However, *A*. *caesareoides*, *A*. *kitamagotake*, and *A*. *rubroflava* differ from *A*. *hemibapha* by having a distinctly umbonate pileus, a much darker and reddish tone in the pileus center and relatively broader basidiospores [3,4,58]. *Amanita subhemibapha*, originally reported from China, differs from *A*. *hemibapha* by having a lighter yellowish tone pileus and relatively broader basidiospores (8.0–11.0 × 6.0–8.0 μm) [4]. According to illustrations of the Thai specimens (Figure 3), most of them are orange-red to lemon yellow at the center of the pileus. This feature was different from the original description of *A. hemibapha* due to the presence of red to orange-red in the center of the pileus. This may be influenced by the phenotypic variability that exists across a wide geographic range. However, the sizes of basidiomata, other macroscopic and microscopic features of the Thai specimens agree well with descriptions of previous studies [3,4,5,10,55,56,57]. Hence, we identify our specimens as *A. hemibapha* using a combination of morphological and molecular data.

#### 3.3.2. ***Amanita pseudoprinceps*** Y.Y. Cui, Q. Cai & Zhu L. Yang, Fungal Divers. 91: 59 (2018) (Figure 4)

*Basidioma* medium-sized to very large. *Pileus* 8.5–16 cm diam., hemispherical, convex to applanate with age, light yellow (4A5–6) to greyish orange (5B3–4) or sometime golden yellow (5B7–8) at center, and becoming yellow white (4A2–3) to white (4A1) towards the margin; universal veil on pileus absent; margin striate (0.1–0.3 R), non-appendiculate; context 9.5–13.5 mm wide, white (1A1), unchanging. *Lamellae* free, crowded, white to cream white (1A1–2); lamellulae truncate. *Stipe* 11.5–17.2 × 1.1–1.9 cm, subcylindrical with slightly tapering upwards and apex slightly expanded, white, covered by minute, white (1A1) fibrous squamules; context fistulose to broadly fistulose, white (1A1), *Bulb* absent. *Universal veil on stipe base* saccate, membranous, up to 7 cm high, white (1A1). *Partial veil* apical, membranous, white (1A1), becoming fragile or disappear with age.

**Figure 4:**
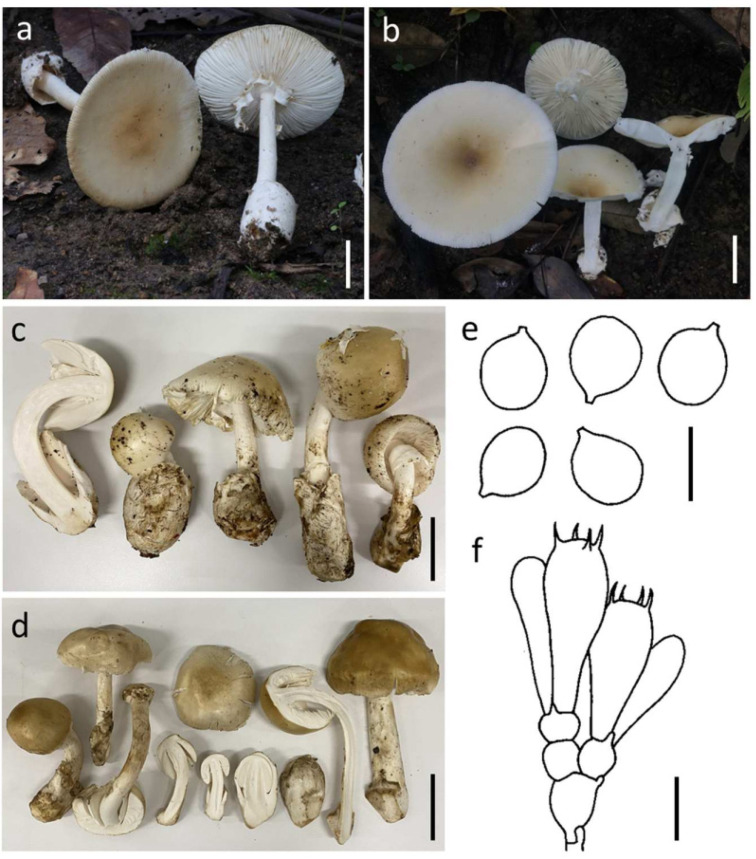
*Amanita pseudoprinceps* SDBR-CMUSTO-2019-472 (**a**), SDBR-CMUNK0775 (**b**), SDBR-CMUNK0853 (**c**) and SDBR-CMUNK0783 (**d**). Basidiomata (**a**–**d**). Basidiospores (**e**). Basidia (**f**). Scale bars: (**a**–**d**) = 5 cm; (**e**) = 10 μm; (**f**) = 15 μm.

*Lamellar trama* bilateral, divergent; mediostratum 40–75 μm wide, filamentous hyphae abundant, 2–12 μm wide; fusiform to ellipsoidal inflated cells 85–213 × 12–26 μm; vascular hyphae scarce to locally abundant. *Subhymenium* 30–50 μm thick in 2–3 layers, with subglobose to ellipsoidal or irregular cells, 12–33 × 10–26 μm. *Basidia* 36–53 × 12–18 μm, clavate, 4-spored with sterigmata 3–6 μm long; clamps present at base. *Basidiospores* (9.0–) 9.5–12.5 (–13.0) × (8.0–) 8.5–12.0 (–12.5) μm, Q = 1.00–1.20 (1.22) μm, Qm = 1.10 ± 0.06, globose to subglobose, sometimes broadly ellipsoid, inamyloid, hyaline, thin-walled, smooth; apiculus small. *Lamellar edge* sterile; filamentous hyphae 2–6 μm wide, hyaline, thin-walled; inflated cells, with subglobose to ellipsoidal, 12–35 × 8–34 μm, single and terminal or in chains of 2–3, hyaline, thin-walled. *Pileipellis* 85–160 μm thick; 2-layered, upper layer 45–80 μm thick, filamentous hyphae 2–5 μm wide, gelatinized, branching, thin-walled, hyaline; lower layer 40–80 μm thick, filamentous hyphae 2–8 (–15) μm wide, branching, thin-walled, hyaline to light yellow; vascular hyphae rare. *Inner surface of universal veil on stipe base* filamentous hyphae dominant 1–8 μm wide, hyaline to light yellow, thin-walled, branching; inflated cells, with subglobose, fusiform to clavate, 50–93 × 15–52 μm, hyaline, thin-walled, mostly terminal or sometimes in chains of 2–3; vascular hyphae rare. *Outer surface of universal veil on stipe base* similar to structure of inner part, but presenting more abundant inflated cells. *Stipe trama* longitudinally acrophysalidic; filamentous, undifferentiated hyphae 2–7 μm wide, thin-walled, frequently branching; acrophysalides 100–233 × 23–45 μm, thin-walled; vascular hyphae rare. *Partial veil* filamentous hyphae very abundant, 1–7 μm wide, hyaline, thin-walled; inflated cells scarce to locally anundant, globose, subglobose to ellipsoidal, 12–70 × 12–35 μm, hyaline to light yellow, thin-walled; vascular hyphae rare. *Clamp connections* present in all tissues of basidioma.

Habitat: Solitary to scattered on soil in tropical deciduous forests dominated by *Dipterocarpus* and *Shorea*. 

Distribution: known from China [4] and Thailand (this study).

Specimens examined: Thailand, Chiang Mai Province, Mae Taeng District, 19°05′38.2″ N 98°52′44.4″ E, alt. 1105 m, 7 August 2019, Yuan S.L., STO-2019-395 (SDBR-CMUSTO-2019-395); Yuan S.L., STO-2019-397 (SDBR-CMUSTO-2019-397); 19°07′45.0″ N 98°45′51.0″ E, alt. 1421 m, 9 August 2019, Yuan S.L., STO-2019-470 (SDBR-CMUSTO-2019-470); Yuan S.L., STO-2019-472 (SDBR-CMUSTO-2019-472). Doi Saket District, 18°53′2″ N 99°9′17″ E, alt. 343 m, 26 July 2020, Kumla J. and. Suwannarach N., CMUNK0783 (SDBR-CMUNK0783), 2 August 2022, Kumla J. and. Suwannarach N., CMUNK0853 (SDBR-CMUNK0853); Lumphun Province, Mae Tha District, 18°27′41″ N 99°10′30″ E, alt. 427 m, 25 July 2020, Kumla J. and. Suwannarach N., CMUNK0775 (SDBR-CMUNK0775).

Remarks: Morphologically, *A. pseudoprinceps* resembles *A. princeps* Corner & Bas by having a similar yellowish-brown pileus and margin striates (about 0.2–0.3 R). However, *A. princeps* presents the larger basidiomata, as well as an outer layer of volval remnants on the stipe cracks and peels in pale buff thin patches [4,54,59]. According to the phylogenetic analysis, our seven samples cluster together with three other samples of *A. pseudoprinceps* and form a well-supported clade that presents a sister clade with *A. aporema* Boedijn. Meanwhile, these two species possess a similar brown tone pileus. However, *A. aporema* has a smaller (6–10 cm) but much darker pileus, as well as obviously longer margin striates (0.5–0.6 R) [4,54,60]. 

#### 3.3.3. ***Amanita rubromarginata*** Har. Takah., Mycoscience 45: 372 (2004) (Figure 5)

*Basidioma* medium-sized to large. *Pileus* 6.0–10.0 cm diam., convex to plano-convex with the center depressed, reddish orange (7B7) over disk, or sometime orange red (8B7–8) at center and becoming light orange (5A4–5) towards the margin; universal veil on pileus absent; margin striate (0.4–0.5 R), non-appendiculate; context 4.5–8.0 mm wide, yellowish white (3A2), unchanging. *Lamellae* free, crowded, pale yellow to light yellow (4A3–4), with lamellar edges reddish orange (7B7–8); lamellulae truncate. *Stipe* 13.7–20.0 × 1.0–1.8 cm, subcylindrical with slightly tapering upwards, yellow (3A6–7), densely covered by reddish yellow to deep yellow (4A7–8) squamules; context broadly fistulose, yellowish white (3A2) to white (3A1). *Bulb* absent. *Universal veil on stipe base* saccate, membranous, up to 5 cm high, white (1A1). *Partial veil* subapical to apical, membranous, dark orange (5A7–8) to orange (6A6–7).

**Figure 5:**
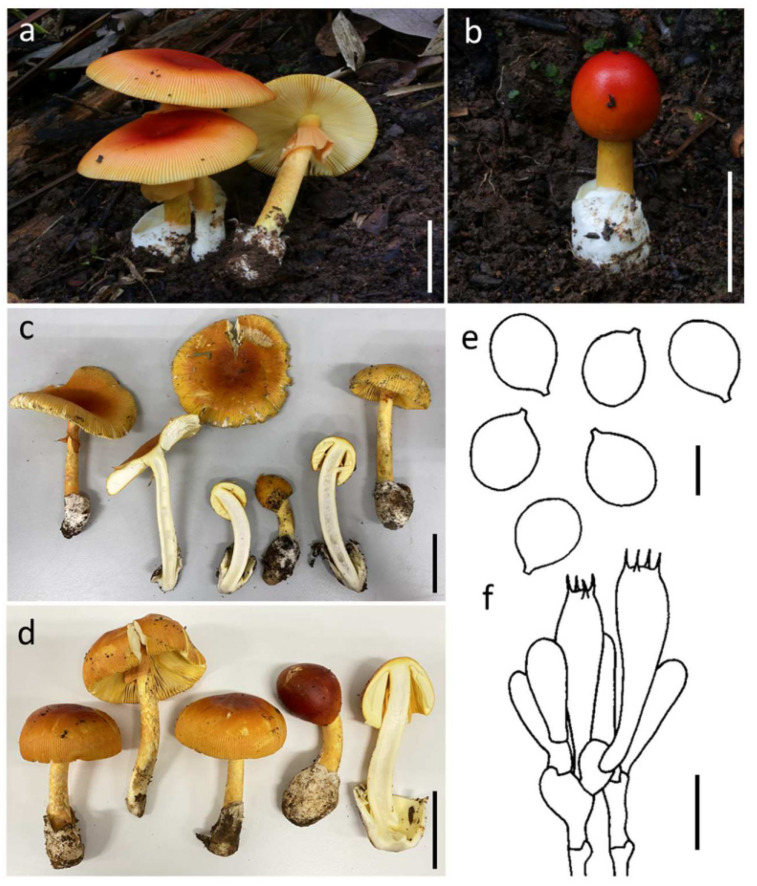
*Amanita rubromarginata* SDBR-CMUSTO-2019-451 (**a**), SDBR-CMUSTO-2019-452 (**b**), SDBR-CMUNK0780 (**c**) and SDBR-CMUNK0854 (**d**). Basidiomata (**a**–**d**). Basidiospores (**e**). Basidia (**f**). Scale bars: (**a**–**d**) = 5 cm; (**e**) = 5 μm; (**f**) = 15 μm.

*Lamellar trama* bilateral, divergent; mediostratum 20–25 μm wide, filamentous hyphae abundant, 2–11 μm wide; fusiform to ellipsoidal inflated cells 60–153 × 15–27 μm; vascular hyphae scarce. *Subhymenium* 25–30 μm thick in 1–3 layers, with subglobose to ellipsoidal or irregular cells, 8–18 × 5–13 μm. *Basidia* 32–46 × 8–13 μm, clavate, 4-spored with sterigmata 3–4 μm long; clamps present at base. *Basidiospores* 7.0–9.5 (–10.0) × 6.0–7.0 (–8.0) μm, Q = (1.08–) 1.13–1.50 μm, Qm = 1.28 ± 0.11, subglobose to broadly ellipsoid or ellipsoid, inamyloid, hyaline, thin-walled, smooth; apiculus small. *Lamellar edge* sterile; filamentous hyphae 3–7 μm wide, hyaline, thin-walled; inflated cells, with globose, pyriform to clavate, 15–46 × 12–27 μm, hyaline, thin-walled. *Pileipellis* 80–130 μm thick; 2-layered, upper layer 25–40 μm thick, filamentous hyphae 1–6 μm wide, gelatinized, branching, thin-walled, hyaline; lower layer 50–100 μm thick, filamentous hyphae 3–8 μm wide, branching, thin-walled, hyaline to light yellow; vascular hyphae rare. *Inner part of universal veil on stipe base* filamentous hyphae dominant 2–11 μm wide, hyaline to light yellow, thin-walled, branching; inflated cells, with subglobose, ovoid to clavate, 30–72 × 10–70 μm, hyaline, thin-walled; vascular hyphae rare. *Outer surface of universal veil on stipe base* similar to structure of inner part, but presenting more abundant inflated cells. *Stipe trama* longitudinally acrophysalidic; filamentous, undifferentiated hyphae 1–6 μm wide, thin-walled, frequently branching; acrophysalides 150–295 × 33–53 μm, thin-walled; vascular hyphae rare. *Partial veil* filamentous hyphae very abundant, 2–11 μm wide, hyaline, thin-walled; inflated cells scarce to locally abundant, ellipsoidal to clavate, 45–110 × 10–16 μm, hyaline to light yellow, thin-walled; vascular hyphae rare. *Clamp connections* present in all tissues of basidioma.

Habitat: Solitary to scattered on soil in tropical deciduous forests dominated by *Dipterocarpus* and *Shorea*. 

Distribution: known from China [4], Japan [61,62], and Thailand [52], this study.

Specimens examined: Thailand, Chiang Mai Province, Mae Taeng District, 19°06′53.3″ N 98°44′22.7″ E, alt. 1718 m, 8 August 2019, Yuan S. Liu, STO-2019-451 (SDBR-CMUSTO-2019-451); STO-2019-452 (SDBR-CMUSTO-2019-452); Doi Saket District, 18°53′2″ N 99°9′17″ E, alt. 343 m, 2 August 2022, Kumla J. and Suwannarach N., CMUNK0854 (SDBR-CMUNK0854); Lumphun Province, Mae Tha District, 18°27′41″ N 99°10′30″ E, alt. 427 m, 25 July 2020, Kumla J. and Suwannarach N., CMUNK0780 (SDBR-CMUNK0780).

Remarks: Morphologically, *A. rubroflava* is easily confused with *A. rubromarginata*. However, *A. rubroflava* differs from *A. rubromarginata* by having a distinctly umbonate pileus and larger basidiospores (8.0–10.0 × 6.5–8.0 μm) [4]. Phylogenetically, *Amanita javanica* (Corner & Bas) Oda, Tanaka & Tsuda is closely related to *A*. *rubromarginata*. Meanwhile, both these two species share similar characteristics, such as an orange-red tone pileus and reddish yellow squamules covering their stipes. However, *A*. *javanica* has a distinctly umbonate pileus, while *A*. *rubromarginata* does not appear to display this characteristic [4,54,59,62]. 

#### 3.3.4. ***Amanita subhemibapha*** Zhu L. Yang, Y.Y. Cui & Q. Cai, Fungal Divers. 91: 65 (2018) (Figure 6)

*Basidioma* medium-sized to large. *Pileus* 6.0–10.0 cm diam., convex to plano-convex, lacking an umbo at center, purely orange (5B5–8) when young, but becoming orange (5B5–8) at center and yellow (4A6–8) to yellowish (3A3–6) at margin when mature; universal veil on pileus absent; margin striate (0.25–0.3 R), non-appendiculate; context 4.5–5.0 mm wide, yellow (4A6–8) to yellowish (3A3–6), unchanging. *Lamellae* free, crowded, white (1A1) to cream (1A4–6), with lamellar edges yellow (4A6–8); lamellulae truncate. *Stipe* 5–15 × 0.7–1.5 cm, subcylindrical with slightly tapering upwards, with apex slightly expanded, yellow (4A6–8) to orange (5B5–8), with its surface covered with concolorous, snakeskin-shaped squamules; context white (1A1), hollow in center. *Bulb* absent. *Universal veil on stipe base* saccate, membranous, up to 5 cm high 3, white (1A1). *Partial veil* apical to subapical, yellow (4A6–8) to orange (5B5–8). 

**Figure 6:**
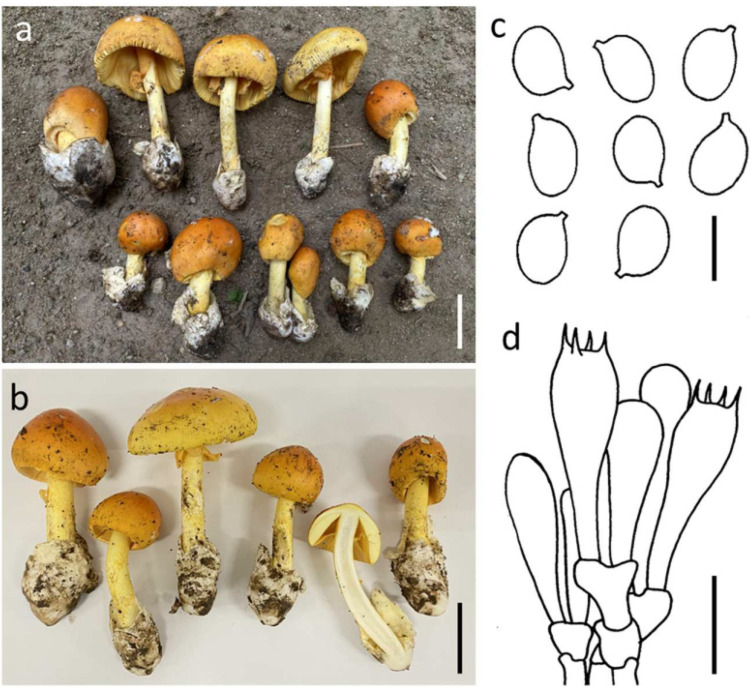
*Amanita subhemibapha* SDBR-CMU0781 (**a**) and SDBR-CMU0855 (**b**). Basidiomata (**a**,**b**). Basidiospores (**c**). Basidia (**d**). Scale bars: (**a**,**d**) = 5 cm; (**c**) = 10 μm; (**d**) = 15 μm.

*Lamellar trama* bilateral, divergent; mediostratum 25–70 μm wide, filamentous hyphae abundant, 2–7 μm wide; ellipsoid, fusiform to clavate inflated cells 30–80 × 10–27 μm; vascular hyphae scarce. *Subhymenium* 30–50 μm thick in 2–3 layers, with subglobose to ellipsoid cells, 10–25 × 8–20 μm. *Basidia* 40–50 × 9–12 μm, clavate, 4-spored with sterigmata 3–5 μm long; clamps present at base. *Basidiospores* (7.0–) 8.0–11.0 × 6.5–8.5 (–9.0) μm, Q = 1.15–1.53 (–1.65), Qm = 1.34 ± 0.08, broadly ellipsoid to ellipsoid, inamyloid, hyaline, thin-walled, smooth; apiculus small. *Lamellar edge* sterile; filamentous hyphae 2–4 μm wide, hyaline, thin-walled; inflated cells, with subglobose to ellipsoid or sphaeropedunculate, 8–45 × 8–20 μm, single and terminal or in chains of 2–3, hyaline, thin-walled. *Pileipellis* 90–170 μm thick; 2-layered, upper layer 30–145 μm thick, filamentous hyphae 2–5 μm wide, gelatinized, branching, thin-walled, hyaline; lower layer 30–55 μm thick, filamentous hyphae 3–8 (–10) μm wide, branching, thin-walled, hyaline to light yellow; vascular hyphae scarce. *Inner surface of universal veil on stipe base* filamentous hyphae dominant 2–10 μm wide, hyaline to light yellow, thin-walled, branching; inflated cells, with subglobose, fusiform to ellipsoid, 55–100 × 20–70 μm, hyaline, thin-walled, mostly terminal or sometimes in chains of 2–3; vascular hyphae rare. *Outer surface of universal veil on stipe base* similar to structure of inner part, but presenting more abundant inflated cells. *Stipe trama* longitudinally acrophysalidic; filamentous, undifferentiated hyphae 2–10 (–15) μm wide, thin-walled, frequently branching; acrophysalides 60–260 × 25–65 μm, thin-walled; vascular hyphae rare. *Partial veil* filamentous hyphae very abundant, 2–10 μm wide, hyaline, thin-walled; inflated cells scarce to locally abundant, subglobose, fusiform to clavate, 20–100 × 10–35 μm, hyaline to light yellow, thin-walled; vascular hyphae rare. *Clamp connections* present in all tissues of basidioma.

Habitat: Solitary to scattered on soil in subtropical broad-leaved or mixed forests with *Dipterocarpaceae*, *Fagaceae*, and *Pinaceae*.

Distribution: known from China [4] and Thailand (this study).

Specimens examined: Thailand, Chiang Mai Province, Doi Saket District, 18°53′2″ N 99°9′17″ E, alt. 343 m, 26 July 2021, Kumla J. and Suwannarach N., CMUNK0804 (SDBR-CMUNK0804); 18°53′2″ N 99°9′17″ E, alt. 343 m, 2 August 2022, Kumla J. and Suwannarach N., CMUNK0855 (SDBR-CMUNK0855); Lumphun Province, Mueang District, Chiang Mai University Haripunchai Campus, 18°32′34″ N 99°9′231″ E, alt. 450 m, 25, July, 2020, Suwannarach N., CMUNK0781 (SDBR-CMUNK0781); Mae Tha District, 18°27′41″ N 99°10′30″ E, alt. 427 m, 27 August 2020, Kumla J. and Suwannarach N., CMUNK0735 (SDBR-CMUNK0735).

Remarks: Morphologically, *A. subhemibapha* is easily confused with *A. hemibapha*, *A. javanica* and *A*. *kitamagotake*. Morphological comparisons of *A. hemibapha* and *A. subhemibapha* have been included in our remarks pertaining to *A. hemibapha*. *Amanita javanica* differs from *A*. *subhemibapha* by having a broadly umbonate and much darker yellow tone in the center of the pileus, longer tuberculate striates (0.4–0.5 R) on the margins and smaller basidiospores (7.5–9.0 × 5.8–7.0 μm) [54]. Alternatively, *A. kitamagotake* differs from *A*. *subhemibapha* by having an umbonate pileus and narrower basidiospores (9.0–13.5 × 6.5–8.5 μm) [58]. Based on multigene phylogeny, *A*. *subhemibapha* forms a sister clade with *A*. *fuscoflava* Zhu L. Yang, Y.Y. Cui & Q. Cai. However, *A*. *fuscoflava* has a dark brown tone in the pileus center, much longer margin striates (0.5–0.7 R) and relatively narrower basidiospores (8.5–10.5 × 6.0–7.0 μm) [4].

Traditionally, morphological characteristics have been the primary basis for the identification of *Amanita* species [7,8,11,32]. However, identification can be difficult due to the high phenotypic variability that is influenced by differing environmental conditions and geographic distributions. Therefore, it is crucial to identify the *Amanita* species using DNA-based methods. The current classification of the genus *Amanita* is based on combined data on their morphological characteristics and molecular data. Moreover, multi-gene molecular phylogeny has provided researchers with a powerful tool for the identification of the *Amanita* species [4,14,36,52,53,54,58]. In this present study, specimens of the edible *Amanita* species collected in northern Thailand were identified as *A. hemibapha, A. pseudoprinceps, A. rubromarginata,* and *A. subhemibapha* based on morphological characteristics and multi-gene phylogenetic analyses. The results of morphological comparisons of four edible *Amanita* species in this study are presented in Table 3. Morphologically, the color of the pileus and the larger spore size found in *A*. *pseudoprinceps* clearly differentiate it from those other three species. Additionally, the yellow annulus and narrow spores in *A*. *hemibapha* clearly distinguish it from *A*. *rubromarginata* and *A*. *subhemibapha*. Remarkably, *A. rubromarginata* has a redder and more of an orange-red-shaded pileus and annulus than *A*. *subhemibapha*. The multi-gene phylogenetic analysis also supports the determination that *A. hemibapha*, *A. pseudoprinceps*, *A. rubromarginata,* and *A. subhemibapha* are different species. Four *Amanita* species obtained from natural forests, roadsides, and local markets in this study belonged to the *Amanita* section *Caesareae*. This section is a highly regarded edible mushroom in the genus *Amanita* [4,16,17,18,19]. Prior to this study, the toxicological analysis of *A. hemibapha* showed that no amatoxins and phallotoxins had been discovered and that it should be regarded as an edible species [63]. However, further research is required to fully understand the edibility and safety of *A. pseudoprinceps, A. rubromarginata*, and *A. subhemibapha* based on their toxicological studies. As a result, our study should be considerably important and highly valuable in terms of stimulating deeper investigations of edible macrofungi in Thailand. It will also help researchers in understanding the distribution and ecology of *Amanita*.

### 3.4. Nutritional Analysis

A total of six samples of four edible *Amanita* species (namely *A. hemibapha*, *A. pseudoprinceps*, *A. rubromarginata*, and *A. subhemibapha*) obtained in this study have been included in the experiments. In this study, the fruiting bodies of edible *Amanita* were analyzed for their nutritional composition, which included ash, carbohydrate, protein, fat and fiber. The results are presented in Table 4. The results indicate that the protein contents in *A. pseudoprinceps* and *A. subhemibapha* were significantly higher than *A. hemibapha* and *A. rubromarginata*. The highest content of fiber was found in *A. pseudoprinceps*. It was determined that *A. rubromarginata* had the highest ash content. In addition, the carbohydrate content in *A. hemibapha* was significantly higher than the other *Amanita* species. The highest fat content was obtained in *A. rubromarginata*, but this value was not found to be significantly different from the fat content of *A. hemibapha*. These results were consistent with previous studies, which reported that edible wild mushrooms to be natural sources of nutrients for human diets (high-protein and low-fat contents), while the nutritional composition of each mushroom is dependent upon the mushroom species [20,22,64,65]. The amounts of ash, carbohydrate, protein, fat, and fiber of the four edible *Amanita* species in this study were within the ranges mentioned in previous reports of edible *Amanita*. Accordingly, the ash (0.11–11.82% dry weight), carbohydrate (22.16–61.70% dry weight), protein (10.11–45.65% dry weight), fat (0.17–17.52% dry weight) and fiber (1.18–30.30% dry weight) contents were found in various edible *Amanita* species, namely *A. caesarea*, *A. calyptroderma*, *A. fulva*, *A. hemibapha*, *A. princeps*, *A. rubescens*, and *A. zambiana* [66,67,68,69,70,71,72,73,74,75]. When compared to the findings of other previously published reports, the protein content of the *Amanita* species obtained in this study was relatively higher than those of *A. calyptroderma* [75] and *A. loosei* [69]. With regard to the outcomes of this study, this is the first comprehensive report on the nutritional composition of *A. pseudoprinceps*, *A. rubromarginata*, and *A. subhemibapha*.

### 3.5. Determination of Total Phenolic Content

The total phenolic content of each extract of *Amanita* in this study is presented in Table 5. It was found that the total phenolic contents ranged from 0.94–1.62 mg GAE/g dw. The highest value of total phenolic content was found in the extract of *A. pseudoprinceps*, followed by the extracts of *A. subhemibapha* and *A. hemibapha*. The lowest value of total phenolic content was found in the extract of *A. rubromarginata*. Previous findings support the results of this study in that the amount of phenolic contents of edible wild mushrooms varied within different ranges and was dependent upon the various mushroom species [45,76,77,78]. According to our results, the amounts of total phenolic content obtained in this study were within the previously reported ranges of phenolic content found in edible wild mushrooms and varied from 0.39–38.44 mg GAE/g dw [76,77,78,79]. The total phenolic contents in the methanolic extracts of *A. caesarea* [79], *A. fulva* [74], *A. hemibapha* [80], *A. javanica* [81], *A. ovoidea* [82], *A. princeps* [80,81], and *A. zambiana* [73] were reported as 0.64, 0.39, 8.5, 18.01, 0.50, 14.29–16.80 and 8.76 mg GAE/g dw, respectively. Additionally, the total phenolic contents in the ethanolic extracts of *A. javanica* and *A. princeps* were 12.79 and 16.52 mg GAE/g dw, respectively [81]. When compared to the results of previously published reports, the phenolic contents of the ethanolic extracts of *A. hemibapha*, *A. pseudoprinceps*, *A. rubromarginata*, and *A. subhemibapha* obtained in this study have been found to be relatively higher than those of methanolic extracts of *A. caesarea*, *A. fulva* and *A. ovoidea* [74,79,82], while they were relatively lower than extracts of *A. javanica, A. princeps* and *A. zambiana* [73,81]. However, the phenolic content of *A. hemibapha* obtained in this study was lower than that of the previous report of Butkhup et al. [80]. It can be concluded from our experiments that, similarly to the results of previous studies, the total content of phenolic can be influenced by different phenolic compounds found in mushroom extracts, along with the extractability of the different solvents used in the preparation process [45,81,83,84]. According to several previous studies, catechin, *р*-coumaric acid, gallic acid, hydroxycinnamic acid, quercetin, protocatechuic acid, rosmarinic acid, and syringic acid were found to be the major phenolic components in the ethanolic extracts of edible wild mushrooms [45,85,86,87]. Some previous investigations revealed that the Folin–Ciocalteu assay, a method typically used for detection and quantification of total phenolic content, might be unsuited for total phenolic content measurement in complex biological samples due to high interference from various reducing compounds contained in samples [88,89,90]. The effectiveness of the Folin–Ciocalteu assay is also hampered by its limited suitability for some phenolic compounds [89,90]. Therefore, the measurement of total phenolic content in this study will still be assessed using other techniques such as high-performance liquid chromatography (HPLC) or liquid chromatography–mass spectrometer mass spectrometry (LC-MS) for further studies to characterize and identify the phenolic compounds contained in mushroom extracts.

### 3.6. Antioxidant Assay

A single method cannot fully determine the antioxidant activity of mushroom extracts. Thus, in this study, three methods, namely ABTS, DPPH, and FRAP assays, were used to determine the antioxidant activity of the ethanolic extracts of different samples of edible *Amanita* species. The ABTS and DPPH values were determined by evaluating the scavenging abilities of ABTS and DPPH radicals, respectively (by measuring the decrease in ABTS and DPPH radical absorption after exposure to radical scavengers) [91,92]. The FRAP assay was used to measure the conversion of the ferric form (Fe^3+^) to the ferrous form (Fe^2+^) [92]. In this study, the highest values of DPPH activity were observed in the extract of the *A*. *pseudoprinceps*, followed by the extracts of *A. hemibapha* and *A. subhemibapha* (Table 5). The lowest value of DPPH activity was observed in the extract of *A. rubromarginata*. Furthermore, the results indicated that all extracts exhibited positive results in terms of the ABTS and FRAP assays, while the ABTS values varied from 0.56 to 1.00 mg TE/g dw (Table 5). The highest ABTS value was observed in the extract of *A. pseudoprinceps*, followed by the extracts of *A. hemibapha*, *A. subhemibapha*, and *A. rubromarginata*. In the FRAP system, the extract of *A. pseudoprinceps* had significantly higher FRAP values than the extracts from the other samples (Table 5). The results from the ABTS, DPPH, and FRAP assays were similar and demonstrated that the extract of *A. pseudoprinceps* exhibited significantly high antioxidant activity. The lowest level of antioxidant activity was found in the extract of *A. rubromarginata*. According to Pearson correlation (*p* < 0.05), the total phenolic content of mushroom extract samples showed a significant strong positive correlation with DPPH (*r* = 0.975) and FRAP (*r* = 0.948) activities (Table 6). However, the positive correlation between the total phenolic content and ABTS activity (*r* = 0.762) was not statistically significant.

All extracts of the four edible *Amanita* species exhibited antioxidant activities. These results are consistent with those of previous studies which reported that the extracts of wild mushrooms (e.g., genera *Amanita*, *Boletus*, *Cantharellus*, *Lactarius*, and *Russula*) exhibited antioxidant activities that varied according to the mushroom species [45,66,78,80,81,82,83]. Furthermore, recent research has indicated that wild mushrooms contain dietary ingredients that are alternative sources of natural antioxidants [45,77,93]. In this study, *A. pseudoprinceps* exhibited the highest level of antioxidant activity due to the fact that it possesses high total polyphenol content. This determination is supported by the results of previous studies, which reported that high phenolic content is responsible for the high antioxidant activity [45,83,94]. Prior to this present study, the antioxidant activities of *A. caesarea*, *A. calyptroderma*, *A. hemibapha*, *A. javanica*, *A. loosei*, *A. ovidea*, and *A. princeps* have been reported from a variety of assays employing different mechanisms including lipid peroxidation, metal chelation, reducing power and scavenging activity, among others [69,75,79,80,81]. However, variations in the assays themselves, and the results they express, make it difficult to compare the outcomes obtained in this study with those of previous studies.

### 3.7. Determination of α-Glucosidase Inhibitory Activity

Importantly, *α*-glucosidase is one of the key enzymes related to hyperglycemia by leading to an increase in blood glucose levels [95,96]. Therefore, inhibition of the function of this enzyme can reduce and control the risk of hyperglycemia. In this study, the *α*-glucosidase inhibition activity of the extracts of each edible *Amanita* species was investigated in terms of the inhibition percentage. The results were then compared with those of acarbose (anti-diabetic drug). The results then revealed that all extract samples exhibited *α*-glucosidase inhibition activity, while the value of the inhibition percentage varied according to the differences in the extract samples (Table 5). The value of *α*-glucosidase inhibition activity in the extract samples varied from 19.26% to 31.44% inhibition. However, all mushroom extracts were found to be less effective than acarbose, a synthetic standard Inhibitor of *α*-glucosidase (44.06% inhibition at concentration of 1 mg/mL). These results are supported by those of previous studies, which reported that the extracts of certain edible wild mushrooms (e.g., *Amanita*, *Astraeus*, *Boletus*, *Lactarius*, *Phlebopus*, *Russula*, *Suillus*, and *Tylopilus*) have potential as natural *α*-glucosidase inhibitors. Accordingly, the *α*-glucosidase inhibition activity varied from 9.72–78.75% for each different mushroom species [45,97,98]. In this study, the amounts of *α*-glucosidase inhibitory activity obtained in this study were within the ranges reported from previous studies. Compared with the outcomes of a report conducted by Pongkunakorn et al. [97], the *α*-glucosidase inhibitory activity of the methanolic extracts of *A. hemibapha* (19.26 and 20.37%) and *A. rubromarginata* (20.28%) obtained in this study were lower than the *α*-glucosidase inhibitory activity of the water extracts of *A. hemibapha* and *A. princeps*, which were reported at 22.66% and 25.54%, respectively. Interestingly, the *α*-glucosidase inhibitory activity of the methanolic extracts of *A. pseudoprinceps* obtained in this study was higher than the *α*-glucosidase inhibitory activity of the water extracts of both *A. hemibapha* and *A. princeps* [97]. Several previous studies have reported that the use of different solvents resulted in different patterns of active compounds in mushroom extracts, which were related to biological activities including *α*-glucosidase inhibitory activity [83,84,97,98]. Importantly, this study is the first report on the *α*-glucosidase inhibition activities of *A. pseudoprinceps*, *A. rubromarginata*, and *A. subhemibapha*. This study found that the extracts of *A. pseudoprinceps* displayed a high level of *α*-glucosidase inhibition activity over the other extracts, which could be related to their high total phenolic content. Additionally, the total phenolic content of all mushroom extracts and *α*-glucosidase inhibitory activity were shown to be significantly correlated by Pearson correlation (*p* < 0.05) (Table 6). These results were similar to those of previous studies [45,99,100], which revealed that the *α*-glucosidase inhibitory activity of natural substances is strongly correlated with the phenolic compound content. 

## 4. Conclusions

The edible *Amanita* specimens collected in northern Thailand were identified as *A*. *hemibapha*, *A*. *pseudoprinceps*, *A*. *rubromarginata*, and *A*. *subhemibapha* based on the relevant morphological characteristics and multi-gene phylogenetic analyses. These four *Amanita* species were selected for further experiments, wherein their nutritional composition, total phenolic content, antioxidant activities, and *α*-glucosidase inhibitory activities were evaluated. All *Amanita* species were high in protein and carbohydrate but low in fat content. Additionally, the methanolic extracts of these four *Amanita* species contained varied amounts of total phenolic content and exhibited varied results in terms of their antioxidant and *α*-glucosidase inhibitory activities. The highest levels of antioxidant and *α*-glucosidase inhibitory activities were found in the methanolic extract of *A*. *pseudoprinceps*. The findings of this investigation provide valuable information on the nutrient content, total phenolic content, and the antioxidant and *α*-glucosidase inhibitory potential of the edible *Amanita* species found in northern Thailand. Therefore, our results suggest that these four edible *Amanita* species can be representative of an alternative food source. These species are also a good source of natural antioxidants and exhibit potential to naturally inhibit *α*-glucosidase for human health benefits. However, future studies should be implemented to conduct a comprehensive mineral analysis and to identify the phenolic profiles present in each edible *Amanita* species. 

## Figures and Tables

**Table 1:** Sequence information used in the molecular phylogenetic analyses.

*Amanita* Species	Strain/Voucher	Country	GenBank Accession Number			
			ITS	nrLSU	*rpb2*	*tef-1*
*A. alboumbelliformis*	HKAS 83448 ^T^	China	–	MH486635	MH486085	MH508892
*A. alboumbelliformis*	HKAS 100495	China	–	MH486634	MH486084	MH508891
*A. aporema*	FRIM 62674	Malaysia	KU714575	KU714551	KU714593	KU714538
*A. arkansana*	RET-139-10	USA	JX844675	KF877195	KF877036	KP724416
*A. caesarea*	HKAS 96166	Italy	MH508283	MH486418	MH485898	MH508705
*A. caesarea*	RET-036-2	Italy	JX844687	KF877205	KF877042	KP724491
*A. caesareoides*	HKAS92017	China	MH508286	MH486422	MH485902	MH508709
*A. caesareoides*	HKAS71021	Japan	MH508284	MH486419	MH485899	MH508706
*A. chepangiana*	HKAS 56718	China	KU714569	KU714545	KU714588	KU714534
*A. egregia*	RET 136-7	Australia	JX844707	KF877227	KF877052	KF877119
*A. fuscoflava*	HKAS 59800 ^T^	China	MH508372	MH486557	MH486023	MH508827
*A. hemibapha*	RET 342-8	India	–	KF877233	KF877055	KF877124
*A. hemibapha*	SDBR-CMUNK0776	Thailand	OQ199032	OQ187796	OQ200073	OQ200092
*A. hemibapha*	SDBR-CMUNK0819	Thailand	OQ199033	OQ187797	OQ200074	OQ200093
*A. hemibapha*	SDBR-CMUNK0857	Thailand	OQ199034	OQ187798	OQ200075	OQ200094
*A. hemibapha*	SDBR-CMUSTO-2019-477	Thailand	OQ199035	OQ187799	OQ200076	OQ200095
*A. hunanensis*	HKAS 100632	China	MH508396	MH486588	MH486050	MH508856
*A. incarnatifolia*	HKAS 100601	China	MH508403	MH486597	MH486059	MH508865
*A. jacksonii*	RET 109-4	USA	–	KF877247	KF877064	KP724551
*A. jacksonii*	Wolf2183	USA	–	MH486606	MH486063	MH508872
*A. javanica*	FRIM 61503	Malaysia	KU714572	KU714548	–	KU714536
*A. kitamagotake*	HKAS 100826B	China	MW258868	MW258920	–	MW324494
*A. kitamagotake*	HKAS 107309B	China	MW258874	MW258921	–	MW324495
*A. longistriata*	HKAS 68331	China	MH508428	MH486631	MH486081	MH508888
*A. mafingensis*	RET 348-8	Zambia	JX844729	KF877259	–	KF877148
*A. mafingensis*	H 7002971	Tanzania	JF710834	JF710802	–	JF710822
*A. princeps*	FRIM 62849	Malaysia	KU714576	KU714552	KU714594	KU714539
*A. princeps*	HKAS 60269	China	–	MH486766	MH486184	MH508993
*A. pseudoprinceps*	HKAS 97523 ^T^	China	MH508527	MH486788	MH486202	–
*A. pseudoprinceps*	HKAS 97642	China	–	MH486789	MH486203	MH509015
*A. pseudoprinceps*	HKAS 97520	China	MH508526	MH486787	MH486201	–
*A. pseudoprinceps*	SDBR-CMUNK0775	Thailand	OQ199036	OQ187800	OQ200077	OQ200096
*A. pseudoprinceps*	SDBR-CMUNK0783	Thailand	OQ199037	OQ187801	OQ200078	OQ200097
*A. pseudoprinceps*	SDBR-CMUNK0853	Thailand	OQ199038	OQ187802	OQ200079	OQ200098
*A. pseudoprinceps*	SDBR-CMUSTO-2019-395	Thailand	–	OQ187803	OQ200080	OQ200099
*A. pseudoprinceps*	SDBR-CMUSTO-2019-397	Thailand	OQ199039	OQ187804	OQ200081	OQ200100
*A. pseudoprinceps*	SDBR-CMUSTO-2019-470	Thailand	OQ199040	–	OQ200082	OQ200101
*A. pseudoprinceps*	SDBR-CMUSTO-2019-472	Thailand	–	OQ187805	OQ200083	OQ200102
*A. ristichii*	RET 124-10 ^T^	USA	JX844737	KF877277	–	–
*A. ristichii*	RET 096-1	Canada	JX844738	JX844738	KF877075	KF877162
*A. rubroflava*	HKAS 83089 ^T^	China	MH508568	MH486827	MH486238	MH509054
*A. rubroflava*	HKAS 83649	China	MH508569	MH486828	MH486239	MH509055
*A. rubromarginata*	RET 383-1 ^T^	Japan	JX844739	KF877279	–	KF877164
*A. rubromarginata*	MFLU 15-01420	Thailand	KU904822	KU877538	–	–
*A. rubromarginata*	HKAS89018	China	MH508573	MH486832	MH486243	MH509059
*A. rubromarginata*	SDBR-CMUNK0780	Thailand	OQ199041	OQ187806	OQ200084	OQ200103
*A. rubromarginata*	SDBR-CMUNK0854	Thailand	OQ199042	OQ187807	OQ200085	OQ200104
*A. rubromarginata*	SDBR-CMUSTO-2019-451	Thailand	OQ199043	OQ187808	OQ200086	OQ200105
*A. rubromarginata*	SDBR-CMUSTO-2019-452	Thailand	OQ199044	OQ187809	OQ200087	OQ200106
*A. similis*	FRIM 3740	Malaysia	KU714566	JF710796	–	KU714531
*A. similis*	TFM-M-P934	Indonesia	KU714568	JF710798	–	KU714533
*A. subhemibapha*	HKAS 96847 ^T^	China	–	MH486906	MH486307	MH509127
*A. subhemibapha*	HKAS97518	China	MH508621	MH486907	MH486308	–
*A. subhemibapha*	SDBR-CMUNK0735	Thailand	OQ199045	OQ187810	OQ200088	OQ200107
*A. subhemibapha*	SDBR-CMUNK0781	Thailand	OQ199046	OQ187811	OQ200089	OQ200108
*A. subhemibapha*	SDBR-CMUNK0804	Thailand	OQ199047	OQ187812	OQ200090	OQ200109
*A. subhemibapha*	SDBR-CMUNK0855	Thailand	OQ199048	OQ187813	OQ200091	OQ200110
*A. torrendii*	HKAS 59739	Spain	KU714578	KU714555	KU714591	KU714540
*A. yuaniana*	HKAS 58807	China	MH508653	MH486954	MH486347	MH509174
*A. yuaniana*	HKAS 68662	China	MH508654	MH486957	MH486350	MH509177
*A. zambiana*	De Kesel 3227	Benin	–	KF877307	KF877093	–
*A. zambiana*	RET 261-3	Burundi	–	KF877311	KF877096	KF877193
*A. retenta*	HKAS 70020 ^T^	China	MH508543	MH486802	MH486215	MH509028
*A. shennongjiana*	HKAS 75553 ^T^	China	MH508590	MH486862	MH486270	MH509085

Superscript “^T^” represents type species. “–” represents the absence of sequence data in GenBank database.

**Table 2:** The initial identification and sources of edible *Amanita* obtained in this study.

Intitial Identification	Source	No. of Collection	Specimen Voucher SDBR-CMU
*A. hemibapha*	Natural forest	2	STO-2019-477 and NK0776
	Roadside market	2	NK0819 and NK0857
*A. pseudoprinceps*	Natural forest	5	STO-2019-395, STO-2019-397, STO-2019-470, STO-2019-472, and NK0775
	Roadside market	2	NK0783 and NK0853
*A. rubromarginata*	Natural forest	2	STO-2019-451 and STO-2019-452
	Roadside market	2	NK0780 and NK0854
*A. subhemibapha*	Natural forest	1	NK0781
	Roadside market	3	NK0735, NK0804, and NK0855

**Table 3:** Comparison of morphological characteristics of edible *Amanita* species obtained in this study.

*Amanita* Species	Pileus	Annulus	Basidia (μm)	Basidiospores (μm)
*A. hemibapha*	6–12 cm diam., orange to yellow at center, and yellow to pale yellow at margin	Yellow	32–50 × 8–12	8.0–12.0 × 5.5–7.0
*A. pseudoprinceps*	8.5–16 cm diam., brownish, yellow-brown to brown at center, and cream to white towards margin	White to cream	36–53 × 12–18	9.0–13.0 × 8.0–12.5
*A. rubromarginata*	6–10 cm diam., red to orange-red at center, becoming reddish orange, orange-yellow to yellow towards margin	Reddish to orange-red	32–46 × 8–13	7.0–10.0 × 6.0–8.0
*A. subhemibapha*	5–10 cm diam., orange at center, and yellow to yellowish at margin	Orange to yellow	40–50 × 9–12	7.0–11.0 × 6.5–9.0

**Table 4:** Nutritional value on a dry basis of different edible *Amanita* species in this study.

*Amanita* Species/ Specimen Voucher SDBR	Nutritional Value (% Dry Weight) *				
	Ash	Carbohydrate	Fat	Fiber	Protein
*A. hemibapha*/CMUNK0776	14.39 ± 0.16 b	34.67 ± 0.22 a	9.94 ± 0.44 a	10.03 ± 0.43 b	23.30 ± 0.40 c
*A. hemibapha*/CMUNK0857	14.10 ± 0.22 b	35.17 ± 0.38 a	9.71 ± 0.28 a	9.13 ± 0.25 c	24.37 ± 0.52 c
*A. pseudoprinceps*/CMUNK0770	12.29 ± 0.23 c	30.10 ± 0.26 d	6.05 ± 0.42 c	12.11 ± 0.50 a	27.97 ± 0.43 a
*A. pseudoprinceps*/CMUNK0853	12.11 ± 0.61 c	30.03 ± 0.64 d	6.11 ± 0.06 c	12.29 ± 0.38 a	28.07 ± 0.59 a
*A. rubromarginata*/CMUNK0780	17.84 ± 0.65 a	31.45 ± 0.26 c	10.24 ± 0.81 a	7.75 ± 0.13 e	26.88 ± 0.19 b
*A. subhemibapha*/CMUNK0855	11.99 ± 0.44 c	33.84 ± 0.16 b	9.30 ± 0.08 b	8.74 ± 0.51 d	27.87 ± 0.67 a

* Results are expressed as mean ± standard deviation. According to Tukey’s test (*p* < 0.05), distinct letters within the same column are regarded as statistically different.

**Table 5:** Total phenolic content, antioxidant and *α*-glucosidase inhibitory activities of different edible *Amanita* species in this study.

*Amanita* Species/ Specimen Voucher SDBR	TPC (mg GAE/g dw)	DPPH Assay (mg TE/g dw)	ABTS Assay (mg TE/g dw)	FRAP Assay (mg TE/g dw)	AGI (% Inhibition)
*A. hemibapha*/CMUNK0776	1.03 ± 0.03 b	0.69 ± 0.01 b	0.87 ± 0.04 c	0.45 ± 0.03 b	20.37 ± 0.99 d
*A. hemibapha*/CMUNK0857	1.07 ± 0.02 b	0.66 ± 0.04 b	0.89 ± 0.02 c	0.49 ± 0.03 b	19.26 ± 0.34 d
*A. pseudoprinceps*/CMUNK0770	1.51 ± 0.03 a	1.54 ± 0.01 a	0.95 ± 0.04 b	0.63 ± 0.02 a	29.14 ± 0.71 b
*A. pseudoprinceps*/CMUNK0853	1.62 ± 0.10 a	1.57 ± 0.05 a	1.00 ± 0.03 a	0.60 ± 0.04 a	31.44 ± 0.71 b
*A. rubromarginata*/CMUNK0780	0.94 ± 0.02 c	0.44 ± 0.12 d	0.56 ± 0.01 e	0.38 ± 0.03 c	20.28 ± 0.23 d
*A. subhemibapha*/CMUNK0855	1.09 ± 0.08 b	0.49 ± 0.02 c	0.70 ± 0.05 d	0.47 ± 0.01 b	23.90 ± 1.10 c
Standard Compound: Acarbose	NT	NT	NT	NT	44.06 ± 0.78 a

TPC = total polyphenol content, AGI = *α*-glucosidase inhibitory assay, and NT = Not Tested. Results are expressed as mean ± standard deviation. According to Tukey’s test (*p* < 0.05), distinct letters within the same column are regarded as statistically different.

**Table 6:** Pearson correlation coefficient (*r*) of the total phenolic content with antioxidant and *α*-glucosidase inhibitory activities of the sample extracts.

Parameter	Antioxidant Activity			AGI Activity
	DPPH Activity	ABTS Activity	FRAP Activity	
Total phenolic content	0.975 *	0.762	0.948 *	0.959 *
*p*-value	*p* < 0.01	*p* = 0.78	*p* = 0.04	*p* = 0.02

“*” indicates a significant positive correlation at a significance level of *p* < 0.05. AGI = *α*-glucosidase inhibitory assay.

## Data Availability

The DNA sequence data obtained from this study have been deposited in GenBank under the accession numbers; ITS (OQ199032–OQ199048); nrLSU (OQ187796– OQ187813); *rpb2* (OQ200073–OQ200091); *tef-1* (OQ200092–OQ200110).
